# Supplementary material for: Quantification of the effect of hemodynamic occlusion in two-photon imaging of mouse cortex
Source: eLife. 2025 May 28;14:RP104914. doi: 10.7554/eLife.104914 (PMC12119086; doi:10.7554/eLife.104914)
Supplement: Supplementary file 1. [file elife-104914-supp1.docx]

Supplementary File 1

| Figure panel | Value compared | Test | P-value | N  (ROIs) | N  (Sites) | N  (Mice) |
| --- | --- | --- | --- | --- | --- | --- |
| 1A | Locomotion onset response in V1 L2/3 | - | - | 2647 | 6 | 6 |
| 1B | Grating onset response in V1 L2/3 | - | - | 2647 | 6 | 6 |
| 1C | Visual response to opto stim light in V1 L2/3 | - | - | 2647 | 6 | 6 |
| 1D | Visuomotor mismatch response in V1 L2/3 | - | - | 2218 | 5 | 5 |
| 1E | Locomotion onset response in V1 L5 | - | - | 1463 | 6 | 6 |
| 1F | Grating onset response in V1 L5 | - | - | 1463 | 6 | 6 |
| 1G | Visual response to opto stim light in V1 L5 | - | - | 1463 | 6 | 6 |
| 1H | Visuomotor mismatch response in V1 L5 | - | - | 947 | 4 | 4 |
| 1I | Locomotion onset response in ACC L2/3 | - | - | 2417 | 6 | 6 |
| 1J | Grating onset response in ACC L2/3 | - | - | 2417 | 6 | 6 |
| 1K | Visual response to opto stim light in ACC L2/3 | - | - | 2417 | 6 | 6 |
| 1L | Visuomotor mismatch response in ACC L2/3 | - | - | 2417 | 6 | 6 |
| 2B-2E | GCaMP6f *vs* GFP | - | - | 15 103, 6527 | 51, 18 | 15, 6 |
| 3A | Fraction of L2/3 neurons in V1 classified responsive to locomotion onsets based on GFP signals | Hierarchical bootstrap | <10^-4^ | 2647 | 6 | 6 |
|  | Fraction of L2/3 neurons in V1 classified responsive to grating onsets based on GFP signals | Hierarchical bootstrap | <10^-4^ | 2647 | 6 | 6 |
|  | Fraction of L2/3 neurons in V1 classified responsive to mismatch onsets based on GFP signals | Hierarchical bootstrap | 0.34 | 2218 | 5 | 5 |
|  | Fraction of L2/3 neurons in V1 classified responsive to locomotion onsets *vs* grating onset based on GFP signals | Hierarchical bootstrap | <10^-4^ | 2647, 2647 | 6,6 | 6,6 |
|  | Fraction of L2/3 neurons in V1 classified responsive to grating onsets *vs* mismatch onset based on GFP signals | Hierarchical bootstrap | <10^-4^ | 2647, 2218 | 6,5 | 6,5 |
|  | Fraction of L2/3 neurons in V1 classified responsive to locomotion onsets *vs* mismatch onset based on GFP signals | Hierarchical bootstrap | <10^-4^ | 2647, 2218 | 6,5 | 6,5 |
| 3B | Fraction of L5 neurons in V1 classified responsive to locomotion onsets based on GFP signals | Hierarchical bootstrap | <10^-4^ | 1463 | 6 | 6 |
|  | Fraction of L5 neurons in V1 classified responsive to grating onsets based on GFP signals | Hierarchical bootstrap | <10^-4^ | 1463 | 6 | 6 |
|  | Fraction of L5 neurons in V1 classified responsive to mismatch onsets based on GFP signals | Hierarchical bootstrap | 0.09 | 947 | 4 | 4 |
|  | Fraction of L5 neurons in V1 classified responsive to locomotion onsets *vs* grating onset based on GFP signals | Hierarchical bootstrap | 0.08 | 1463, 1463 | 6,6 | 6,6 |
|  | Fraction of L5 neurons in V1 classified responsive to grating onsets *vs* mismatch onset based on GFP signals | Hierarchical bootstrap | <10^-4^ | 1463, 947 | 6,4 | 6,4 |
|  | Fraction of L5 neurons in V1 classified responsive to locomotion onsets *vs* mismatch onset based on GFP signals | Hierarchical bootstrap | <10^-4^ | 1463, 947 | 6,4 | 6,4 |
| 3C | Fraction of L2/3 neurons in ACC classified responsive to locomotion onsets based on GFP signals | Hierarchical bootstrap | <10^-4^ | 2417 | 6 | 6 |
|  | Fraction of L2/3 neurons in ACC classified responsive to grating onsets based on GFP signals | Hierarchical bootstrap | 0.18 | 2417 | 6 | 6 |
|  | Fraction of L2/3 neurons in ACC classified responsive to mismatch onsets based on GFP signals | Hierarchical bootstrap | 0.34 | 2417 | 6 | 6 |
|  | Fraction of L2/3 neurons in ACC classified responsive to locomotion onsets *vs* grating onset based on GFP signals | Hierarchical bootstrap | <10^-4^ | 2417, 2417 | 6,6 | 6,6 |
|  | Fraction of L2/3 neurons in ACC classified responsive to grating onsets *vs* mismatch onset based on GFP signals | Hierarchical bootstrap | 0.57 | 2417, 2417 | 6,6 | 6,6 |
|  | Fraction of L2/3 neurons in ACC classified responsive to locomotion onsets *vs* mismatch onset based on GFP signals | Hierarchical bootstrap | <10^-4^ | 2417, 2417 | 6,6 | 6,6 |
| 3D | Fraction of L2/3 neurons in V1 classified responsive to locomotion onsets based on GCaMP6f signals | Hierarchical bootstrap | <10^-4^ | 7971 | 25 | 15 |
|  | Fraction of L2/3 neurons in V1 classified responsive to grating onsets based on GCaMP6f signals | Hierarchical bootstrap | <10^-4^ | 7971 | 25 | 15 |
|  | Fraction of L2/3 neurons in V1 classified responsive to mismatch onsets based on GCaMP6f signals | Hierarchical bootstrap | <10^-4^ | 7653 | 24 | 15 |
|  | Fraction of L2/3 neurons in V1 classified responsive to locomotion onsets *vs* grating onset based on GCaMP6f signals | Hierarchical bootstrap | 0.01 | 7971, 7971 | 25, 25 | 15, 15 |
|  | Fraction of L2/3 neurons in V1 classified responsive to grating onsets *vs* mismatch onset based on GCaMP6f signals | Hierarchical bootstrap | <10^-4^ | 7971, 7653 | 25, 24 | 15, 15 |
|  | Fraction of L2/3 neurons in V1 classified responsive to locomotion onsets *vs* mismatch onset based on GCaMP6f signals | Hierarchical bootstrap | <10^-4^ | 7971, 7653 | 25, 24 | 15, 15 |
| 3E | Fraction of L5 neurons in V1 classified responsive to locomotion onsets based on GCaMP6f signals | Hierarchical bootstrap | <10^-4^ | 7132 | 26 | 14 |
|  | Fraction of L5 neurons in V1 classified responsive to grating onsets based on GCaMP6f signals | Hierarchical bootstrap | <10^-4^ | 7132 | 26 | 14 |
|  | Fraction of L5 neurons in V1 classified responsive to mismatch onsets based on GCaMP6f signals | Hierarchical bootstrap | <10^-3^ | 6812 | 25 | 13 |
|  | Fraction of L5 neurons in V1 classified responsive to locomotion onsets *vs* grating onset based on GCaMP6f signals | Hierarchical bootstrap | 0.19 | 7132, 7132 | 26, 26 | 14, 14 |
|  | Fraction of L5 neurons in V1 classified responsive to grating onsets *vs* mismatch onset based on GCaMP6f signals | Hierarchical bootstrap | <10^-4^ | 7132, 6812 | 26, 25 | 14, 13 |
|  | Fraction of L5 neurons in V1 classified responsive to locomotion onsets *vs* mismatch onset based on GCaMP6f signals | Hierarchical bootstrap | <10^-4^ | 7132, 6812 | 26, 25 | 14, 13 |
| 3F | Fraction of L2/3 neurons in ACC classified responsive to locomotion onsets based on GCaMP signals | Hierarchical bootstrap | <10^-4^ | 2207 | 12 | 7 |
|  | Fraction of L2/3 neurons in ACC classified responsive to mismatch onsets based on GCaMP signals | Hierarchical bootstrap | <10^-4^ | 2207 | 12 | 7 |
|  | Fraction of L2/3 neurons in ACC classified responsive to locomotion onsets *vs* mismatch onset based on GCaMP signals | Hierarchical bootstrap | <10^-4^ | 2207, 2207 | 12,12 | 7,7 |
| 4D-4E | Variance explained in V1 L2/3 neuron GFP signal | - | - | 2647 | 6 | 6 |
| 4D-4E | Variance explained in V1 L5 neuron GFP signal | - | - | 1463 | 6 | 6 |
| 4D-4E | Variance explained in ACC L2/3 neuron GFP signal | - | - | 2417 | 6 | 6 |
| 5A | Locomotion onset response under different conditions in V1 L2/3 | - | - | 2647 | 6 | 6 |
| 5B | Locomotion onset response under different conditions in V1 L5 | - | - | 1463 | 6 | 6 |
| 5C | Locomotion onset response under different conditions in ACC L2/3 | - | - | 2417 | 6 | 6 |
| 6A | Pairwise correlation in GFP signal in V1 L2/3 neurons, stationary *vs* locomoting | Hierarchical bootstrap | <10^-3^ | 2647 | 6 | 6 |
| 6B | Pairwise correlation in GFP signal in V1 L5 neurons, stationary *vs* locomoting | Hierarchical bootstrap | <10^-4^ | 1276 | 6 | 6 |
| 6C | Pairwise correlation in GFP signal in ACC L2/3 neurons, stationary *vs* locomoting | Hierarchical bootstrap | <10^-4^ | 2417 | 6 | 6 |
| 7A | Locomotion onset response in V1 | - | - | 48 | 24 | 11 |
| 7B | Grating onset response in V1 | - | - | 52 | 26 | 12 |
| 7C | Visuomotor mismatch response in V1 | - | - | 48 | 24 | 11 |
| 7D | Locomotion onset response in ACC | - | - | 48 | 24 | 11 |
| 7E | Grating onset response in ACC | - | - | 52 | 26 | 12 |
| 7F | Visuomotor mismatch response in ACC | - | - | 48 | 24 | 11 |
| 8B | Locomotion onset response in GRAB-DA1m in V1 – neuropil and blood vessel ROI response | - | - | 168, 34 | 7, 5 | 7, 5 |
| 8C | Grating onset response in GRAB-DA1m in V1 – neuropil and blood vessel ROI response | - | - | 168, 34 | 7, 5 | 7, 5 |
| 8D | Visuomotor mismatch response in GRAB-DA1m in V1 – neuropil and blood vessel ROI response | - | - | 124, 20 | 5, 3 | 5, 3 |
| 8F | Locomotion onset response in GRAB-5HT1.0 in V1 – neuropil and blood vessel ROI response | - | - | 220, 113 | 10, 10 | 9, 9 |
| 8G | Grating onset response in GRAB-5HT1.0 in V1 – neuropil and blood vessel ROI response | - | - | 205, 101 | 9, 9 | 9, 9 |
| 8H | Visuomotor mismatch response in GRAB-5HT1.0 in V1 – neuropil and blood vessel ROI response | - | - | 193, 101 | 9, 9 | 8, 8 |
| 8J | Locomotion onset response in GRAB-ACh3.0 in V1 – neuropil and blood vessel ROI response | - | - | 178, 118 | 8,8 | 8,8 |
| 8K | Grating onset response in GRAB-ACh3.0 in V1 – neuropil and blood vessel ROI response | - | - | 178, 118 | 8,8 | 8,8 |
| 8L | Visuomotor mismatch response in GRAB-ACh3.0 in V1 – neuropil and blood vessel ROI response | - | - | 178, 118 | 8,8 | 8,8 |
| 9A | Locomotion onset response in GRAB-NE1m in V1 | - | - | 64 | 32 | 5 |
| 9B | Grating onset response in GRAB-NE1m in V1 | - | - | 12 | 6 | 5 |
| 9C | Visuomotor mismatch response in GRAB-NE1m in V1 | - | - | 18 | 9 | 5 |
| 9D | Locomotion onset response in GRAB-NE1m in ACC | - | - | 64 | 32 | 5 |
| 9E | Grating onset response in GRAB-NE1m in ACC | - | - | 12 | 6 | 5 |
| 9F | Visuomotor mismatch response in GRAB-NE1m in ACC | - | - | 18 | 9 | 5 |
| 1-s2A | Locomotion onset response in V1 L2/3 | - | - | 2647 | 6 | 6 |
|  | Grating onset response in V1 L2/3 | - | - | 2647 | 6 | 6 |
|  | Visual response to opto stim light in dark in V1 L2/3 | - | - | 2647 | 6 | 6 |
|  | Visual response to opto stim light in VR in V1 L2/3 | - | - | 2647 | 6 | 6 |
|  | Visuomotor mismatch response in V1 L2/3 | - | - | 2218 | 5 | 5 |
| 1-s2B | Locomotion onset response in V1 L5 | - | - | 1463 | 6 | 6 |
|  | Grating onset response in V1 L5 | - | - | 1463 | 6 | 6 |
|  | Visual response to opto stim light in dark in V1 L5 | - | - | 1463 | 6 | 6 |
|  | Visual response to opto stim light in VR in V1 L5 | - | - | 1463 | 6 | 6 |
|  | Visuomotor mismatch response in V1 L5 | - | - | 947 | 4 | 4 |
| 1-s2C | Locomotion onset response in ACC L2/3 | - | - | 2417 | 6 | 6 |
|  | Grating onset response in ACC L2/3 | - | - | 2417 | 6 | 6 |
|  | Visual response to opto stim light in dark in ACC L2/3 | - | - | 2417 | 6 | 6 |
|  | Visual response to opto stim light in VR in ACC L2/3 | - | - | 2417 | 6 | 6 |
|  | Visuomotor mismatch response in ACC L2/3 | - | - | 2417 | 6 | 6 |
| 1-s3A | Visual flow onset response with GFP in V1 L2/3 | - | - | 2647 | 6 | 6 |
| 1-s3B | Locomotion onset response in dark with GFP in V1 L2/3 | - | - | 2647 | 6 | 6 |
| 1-s3C | Visual flow onset response in closed loop with GFP in V1 L2/3 | - | - | 2647 | 6 | 6 |
| 3-s1 | Average peak GFP response for low vs intermediate GFP expression level | Hierarchical bootstrap | 0.88 | 7076, 6477 | 17, 15 | 6, 6 |
|  | Average peak GFP response for intermediate vs high GFP expression level | Hierarchical bootstrap | 0.36 | 6477, 4857 | 15, 10 | 6, 6 |
|  | Average peak GFP response for low vs high GFP expression level | Hierarchical bootstrap | 0.85 | 7076, 4857 | 17, 10 | 6, 6 |
| 4-s1A | Brain velocity on locomotion onset | - | - | - | 18 | 6 |
| 4-s1B | Brain velocity on grating onset | - | - | - | 18 | 6 |
| 4-s1C | Brain velocity on opto stim light onset in dark | - | - | - | 18 | 6 |
| 4-s1D | Brain velocity on opto stim light onset in VR | - | - | - | 18 | 6 |
| 7-s1A | Locomotion onset response with widefield and no fluorophore in V1 | - | - | 24 | 12 | 6 |
| 7-s1B | Grating onset response with widefield and no fluorophore in V1 | - | - | 24 | 12 | 6 |
| 7-s1C | Visuomotor mismatch response with widefield and no fluorophore in V1 | - | - | 16 | 8 | 4 |
| 7-s1D | Locomotion onset response with widefield and no fluorophore in ACC | - | - | 24 | 12 | 6 |
| 7-s1E | Grating onset response with widefield and no fluorophore in ACC | - | - | 24 | 12 | 6 |
| 7-s1F | Visuomotor mismatch response with widefield and no fluorophore in ACC | - | - | 16 | 8 | 4 |
| 8-s1A | Locomotion onset response in GRAB-DA1m in V1 – neuropil and blood vessel ROI response | - | - | 122, 61 | 7, 7 | 7, 7 |
| 8-s1B | Grating onset response in GRAB- DA1m in V1 – neuropil and blood vessel ROI response | - | - | 122, 61 | 7, 7 | 7, 7 |
| 8-s1C | Visuomotor mismatch response in GRAB- DA1m in V1 – neuropil and blood vessel ROI response | - | - | 80, 50 | 5, 5 | 5, 5 |
| 8-s1D | Locomotion onset response in GRAB-5HT1.0 in ACC – neuropil and blood vessel ROI response | - | - | 123, 79 | 7, 7 | 6, 6 |
| 8-s1E | Grating onset response in GRAB-5HT1.0 in ACC – neuropil and blood vessel ROI response | - | - | 109, 69 | 6, 6 | 6, 6 |
| 8-s1F | Visuomotor mismatch response in GRAB-5HT1.0 in ACC – neuropil and blood vessel ROI response | - | - | 91, 56 | 5, 5 | 5, 5 |
