# Supplementary material for: Quantification of the effect of hemodynamic occlusion in two-photon imaging of mouse cortex
Source: eLife. 2025 May 28;14:RP104914. doi: 10.7554/eLife.104914 (PMC12119086; doi:10.7554/eLife.104914)
Supplement: Supplementary file 2. [file elife-104914-supp2.docx]

Supplementary file 2

| Genotype | Virus | Site of injection | Nbr. Mice | Nbr. ROIs | Figures |  |
| --- | --- | --- | --- | --- | --- | --- |
| C57BL/6 | | AAV2/1-Ef1α-eGFP-WPRE | Primary visual cortex – L2/3 | 6 | 3072 | Figures 1, 2, 3A, 4, 5, 6, Figure 1-figure supplement 1, Figure 1-figure supplement 2, Figure 1-figure supplement 3, Figure 3-figure supplement 1, Figure 4-figure supplement 1, Figure 4-figure supplement 3 |
| C57BL/6 | | AAV2/1-Ef1α-eGFP-WPRE | Primary visual cortex – L5 | 6 | 1710 | Figures 1, 2, 3B, 4, 5, 6, Figure 1-figure supplement 1, Figure 1-figure supplement 2, Figure 1-figure supplement 3, Figure 3-figure supplement 1, Figure 4-figure supplement 1, Figure 4-figure supplement 2, Figure 4-figure supplement 3 |
| C57BL/6 | | AAV2/1-Ef1α-eGFP-WPRE | Anterior cingulate cortex – L2/3 | 6 | 2739 | Figures 1, 2, 3C, 4, 5, 6, Figure 1-figure supplement 1, Figure 1-figure supplement 2, Figure 1-figure supplement 3, Figure 3-figure supplement 1, Figure 4-figure supplement 1, Figure 4-figure supplement 2, Figure 4-figure supplement 3 |
| ChAT-IRES-Cre | | AAV2/1-Ef1α-GCaMP6f-WPRE | Primary visual cortex – L2/3 and L5 | 15 | 15 103 | Figures 2, 3D, 3E |
| C57BL/6 | | AAV-PHP.eB-EF1α-eGFP | Retro-orbital | 8 | 32 | Figure 7 |
| fosGFP | | - | - | 4 | 20 | Figure 7 |
| Tlx3-Cre | | - | - | 6 | 24 | Figure 7-figure supplement 1 |
| ChAT-IRES-Cre | | AAV2/9-hSyn-GRAB-ACh3.0 | Primary visual cortex | 8 | 609 | Figure 8 |
| C57BL/6 | | AAV2/9-hSyn-GRAB-DA1m | Anterior cingulate cortex | 7 | 183 | Figures 8, Figure 8-figure supplement 1 |
|  |  |  | Primary visual cortex | 7 | 202 |  |
| C57BL/6 | | AAV2/9-hSyn-GRAB-5HT1.0 | Anterior cingulate cortex | 9 | 202 | Figures 8, Figure 8-figure supplement 1 |
|  |  |  | Primary visual cortex | 6 | 333 |  |
| C57BL/6 | | AAV-PHP.eB-hSyn-GRAB-NE1m | Retro-orbital | 5 | 156 | Figure 9 |
